# Shared Genetic Architecture Between Atopic Dermatitis and Autoimmune Diseases

**DOI:** 10.3390/ijms26189124

**Published:** 2025-09-18

**Authors:** Panagiotis Lazanas, Charalabos Antonatos, Konstantina T. Tsoumani, Argyro Sgourou, Yiannis Vasilopoulos

**Affiliations:** 1 Laboratory of Genetics, Section of Genetics, Cell Biology and Development, Department of Biology, University of Patras, 26504 Patras, Greece; panagiotislazanas2@gmail.com (P.L.); charisantonatos@gmail.com (C.A.); 2 Biology Laboratory, School of Science and Technology, Hellenic Open University, 26504 Patras, Greece; ktsoumani@gmail.com (K.T.T.); sgourou@eap.gr (A.S.)

**Keywords:** atopic dermatitis, vitiligo, inflammatory bowel disease, rheumatoid arthritis, pleiotropy

## Abstract

Atopic dermatitis (AD) and autoimmune diseases exhibit epidemiological comorbidity, yet the shared genetic architecture remains incompletely understood. We investigated the genetic overlap between AD and three autoimmune disorders including inflammatory bowel disease (IBD), rheumatoid arthritis (RA), and vitiligo, leveraging genome-wide association data. Despite modest evidence for global genetic correlations, we found 113 independent pleiotropic loci shared among AD and autoimmune diseases, with 11 displaying a concordant effect across all 3 pairwise comparisons. Gene-set and tissue enrichment analyses evidenced the inflammatory background of pleiotropic associations. Multi-trait colocalization analysis prioritized 22 loci, linking the tissue-specific expression of *DOK2*, *GPR132*, *RERE*, *RERE-AS1*, *SUOX*, *TNFRSF11A*, and *TRAF1* pleiotropic genes with AD risk. Mendelian randomization revealed no causal effect of genetic liability to AD on autoimmune diseases. Nevertheless, genetic liability to IBD increased AD risk, while vitiligo exhibited a protective effect post outlier correction. Our findings provide mechanistic insights into the multimorbidity of atopic dermatitis (AD) and autoimmune diseases, offering additional evidence for the pleiotropic genetic architecture of AD that contributes to systemic immune dysregulation across multiple organ systems.

## 1. Introduction

Atopic dermatitis (AD), also known as eczema or atopic eczema, is one of the most prevalent skin disorders, exhibiting a 2.62% worldwide prevalence and affecting up to 204.5 million individuals [1]. AD is characterized by intense pruritus, chronic or recurrent eczematous lesions, frequently aggravated by bacterial colonization (e.g., *Staphylococcus aureus*), blistering and crusting at early stages, lichenification at later stages, and discomfort in general, leading to psychological complications, sleep loss, and reduced self-esteem [2]. The pathophysiology of AD is driven by complex interactions between epidermal barrier dysfunction, skin microbiome abnormalities, disruption of type 2 immune regulation, and environmental factors [3]. The disease displays significant heritability, estimated at 80% through linkage studies [4]; one of the most established genetic risk factors is loss-of-function mutations in the filaggrin (*FLG*) gene [5], present in 20% of AD patients [6]. Apart from *FLG* mutations, associations have also been observed in the 5q31.1 chromosomal region [7], including genes encoding type 2 cytokines such as IL-4 and IL-13, while most genome-wide significant loci are enriched in inflammatory pathways, highlighting the systemic nature of the disease.

AD is established as the first occurrence of the atopic march, involving the sequential development of other atopic comorbidities including asthma and allergic rhinitis across the lifespan [6]. Observed associations between AD and other atopic diseases indicate the existence of shared genetic features [8], as exemplified through large-scale cross-trait analyses of atopic march, where 6/136 lead variants showed a disease-specific effect [9]. Most shared variants were mapped across cytokine and T cell-mediated pathways modulating type 2 immune responses and epithelial barrier dysregulation, underpinning a pleiotropic inflammatory axis that underlies the development of distinct atopic disorders.

However, studies have also shown that AD is connected to a wide variety of non-atopic diseases, such as ocular, cardiovascular, musculoskeletal, and metabolic comorbidities [10]. We and others have shown that the genetic background of AD exerts a pleiotropic role in the development of comorbidities including neuropsychiatric disorders [11], gastrointestinal diseases [12], and relevant risk factors [13]. Strong multimorbidity associations have also been reported between AD and autoimmune diseases, where patients with AD have an increased risk for both inflammatory skin diseases like vitiligo, as well as systemic autoimmune disorders such as inflammatory bowel disease (IBD) and rheumatoid arthritis (RA) [10,14]. Using population-based cohort studies, individuals with AD aged 40 years or under had a 72% higher risk of developing RA and a 34% increased risk for IBD [15]. Similarly, large-scale data evidence the 2–3 fold increased prevalence of vitiligo in AD patients compared to the general population [14], indicating genetic overlap across skin-specific inflammatory phenotypes [16]. Emerging data further suggest that AD could be worth considering for reclassification, based on stronger associations with the autoimmune system [17], as more than a cutaneous disease. Concurrently, AD shares pharmacotherapies with the above diseases [3,6] of different systemic nature, where Janus kinase (JAK) inhibitors, such as upadacitinib and tofacitinib, originally developed for autoimmune conditions, have further demonstrated clinical efficacy during administration in patients suffering from AD [18,19]. This common therapeutic landscape indicates the existence of biological mechanisms involved in both the cutaneous allergy and the autoimmunity. Multi-trait GWAS data between autoimmune and allergic diseases have already identified several pleiotropic mechanisms at the gene and pathway level, suggesting the importance of genetics-driven comorbidity estimates [17]. Building on these observations, we hypothesize that cross-trait methodological approaches may inform us on some aspects of multimorbidity estimates, uncovering mechanistic insights at the gene and pathway level. Investigation of this pleiotropy can expand our understanding of the heritable components of AD multimorbidity, challenge traditional disease classifications, and provide new insights into pathogenesis, treatment response, and therapeutic repurposing.

**Figure 1:**
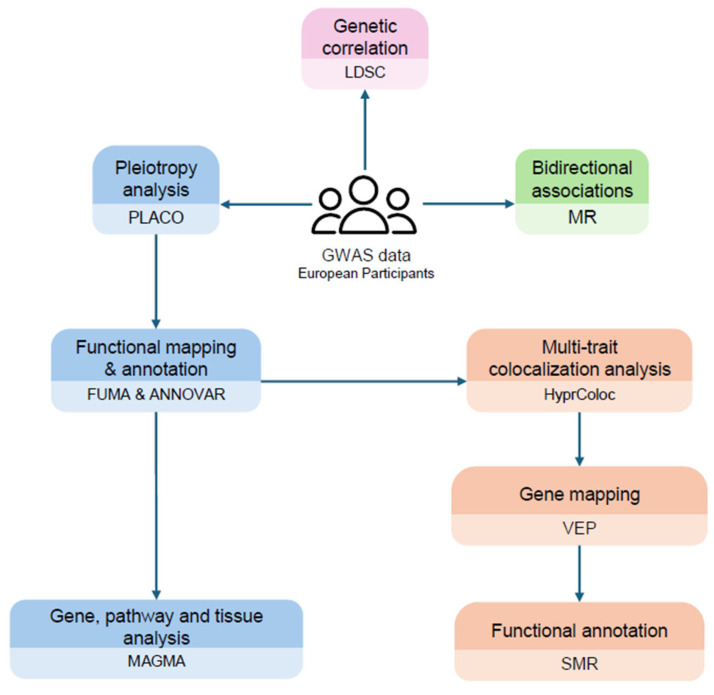
The workflow of the current study.

To test our hypothesis, we investigated the shared genetic architecture between AD and autoimmune diseases. We deliberately selected autoimmune traits spanning mucosal, systemic, and organ-specific autoimmunity to examine whether pleiotropic architecture in AD reflects broad immune dysregulation or is confined to specific pathogenic contexts. Specifically, we focused on IBD as a model of barrier-associated autoimmunity [20], RA as a typical systemic autoimmune disease driven by autoreactive T and B cells [21], and vitiligo as an organ-specific cutaneous autoimmune disease targeting melanocytes [22]. These disorders were prioritized based on strong epidemiological evidence [10,14] and distinct immunopathological mechanisms, thereby maximizing biological contrast and pleiotropic discovery potential. We leveraged single-nucleotide polymorphisms (SNPs) from large-scale GWAS data on Europeans to estimate causality and pleiotropy at the SNP, gene, and pathway level. A framework of our approach is presented in Figure 1.

## 2. Results

### 2.1. Genetic Correlations Between Atopic Dermatitis and Autoimmune Disorders

We gathered available publicly available GWAS data [23] of all selected traits in Europeans (Table 1). Bivariate global genetic correlations identified modest estimates between AD and IBD (r_g_, 95% CI: 0.157, 0.031–0.283, *p*-value = 0.014), while vitiligo (r_g_, 95% CI: 0.026, −0.09–0.143, *p*-value = 0.658), and RA (r_g_, 95% CI: −0.0096, −0.127–0.107, *p*-value = 0.872) estimates overlapped the null.

### 2.2. Pleiotropic Loci Between Atopic Dermatitis and Autoimmune Disorders

**Figure 2:**
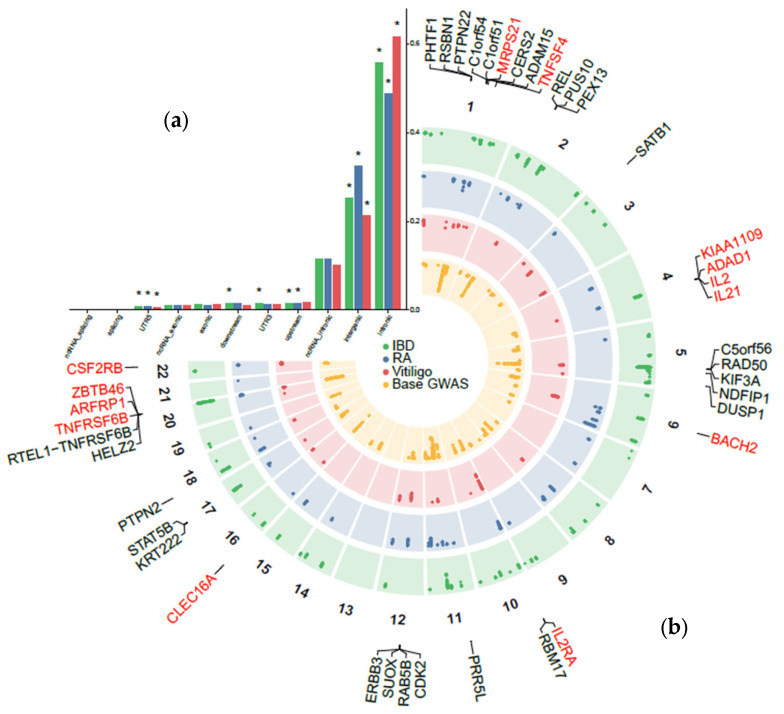
Pleiotropic architecture of atopic dermatitis and autoimmune diseases. (**a**) Functional enrichment analysis of genome-wide significant pleiotropic variants through ANNOVAR. A star sign “*” indicates significant enrichment results. (**b**) Circular Manhattan plot of genome-wide significant (GWS) pleiotropic variants between atopic dermatitis and autoimmune diseases. The inner layer corresponds to the base GWAS GWS variants in atopic dermatitis [7]. The outer layers correspond to GWS variants in vitiligo, rheumatoid arthritis, and inflammatory bowel disease, respectively. The outer layer corresponds to shared genes identified from the MAGMA gene-level analysis (Table S2). Black-colored labels refer to genes shared among any two traits, while red-colored labels refer to genes shared across all three comparisons. Abbreviations: IBD, inflammatory bowel disease; RA, rheumatoid arthritis; GWAS, genome-wide association study.

Pairwise pleiotropic analyses identified 3250 pleiotropic SNPs between AD and IBD, mapped to 70 pleiotropic loci using the functional mapping and annotation of genome-wide association studies (FUMA) web platform. In the AD–RA comparison, 1533 pleiotropic variants were mapped to 50 independent loci, while AD–vitiligo displayed the lowest amount of genome-wide significant (GWS) variants (*n* = 1165), mapped to 31 risk loci (Figure 2; Table S1). The total number of independent pleiotropic loci was 113 (Figure 2b). In line with the modest genetic correlation estimates previously recorded, we observed a mixed direction pattern between lead variants in the AD–IBD pairwise comparison, where 41/70 (58.57%) lead variants showed concordant effects (Figure S1). Similar percentages were derived for RA (19/50, 38%; Figure S2) and vitiligo (7/31, 22.58%; Figure S3).

Functional enrichment analysis performed with annotate variation (ANNOVAR) showed that most pleiotropic variants were mapped in intronic regions (Figure 2a). In particular, variants for vitiligo were significantly localized in intronic regions, representing 61.6% of the total GWS SNPs, with similar estimates in IBD (55.6%) and RA (48.6%; Figure 2a). Next, 32.4% of RA variants were in intergenic regions, 25.2% of IBD, and 21.2% for vitiligo. Statistical significance was also achieved for upstream variation in RA (1.6%) and IBD (1.4%), for UTR3 in IBD only (1.5%), and the UTR3 region for all traits (IBD: 0.8%, RA: 0.9%, vitiligo: 0.6%). These findings align with previous observations where GWS variation is mostly mapped in non-coding regions [27], suggesting strong regulatory activity.

A large number of pleiotropic loci were shared among all pairwise comparisons. Specifically, 41/113 (36%) independent pleiotropic loci were shared across any 2 traits, corresponding to 15.7% for IBD (11/70), 38% for RA (19/50), and 35.4% for vitiligo (11/31). Out of all pleiotropic loci identified, 11/113 (9%) were shared across all 3 traits, corresponding to 15.7% for IBD (11/70), 22% for RA (11/50), and 35.4% for vitiligo (11/31).

### 2.3. Prioritization of Candidate Pleiotropic Genes and Pathways with Tissue Specificity

Gene prioritization analyses through the multi-marker analysis of genomic annotation (MAGMA) v1.10 resulted in 243 significant gene-trait associations for all pairwise comparisons, reduced to 188 unique (Table S2). As expected, some genes (40/188, 21.2%) met more than two pairwise associations with autoimmune diseases. Specifically, 27 genes were associated with at least 2 pairwise comparisons, with the exemplars of *PTPN22*, *REL*, *KIF3A*, *PPR5L*, and *SUOX*, all of which have been previously associated with AD [7] (Figure 2b). Thirteen (13) genes were further prioritized across all comparisons, mapped in previous risk loci for AD including *MRPS21* and *TNFSF4* in chromosome 1, *KIAA1109*, *ADAD1*, *IL1* and *IL21* in chromosome 4, *BACH2* in chromosome 6, *IL2RA* in chromosome 10, *CLEC16A* in chromosome 16, *ZBTB46*, *ARFRP1* and *TNFRSF6B* in chromosome 20 and *CSF2RB* in chromosome 22 (Figure S4).

Competitive gene-set analysis reported 261 unique enriched terms, including 233 gene sets among AD–IBD, 114 in the AD–RA and 47 in the AD–vitiligo pairwise comparisons (Table S3). Several terms (166) showed a comparison-specific enrichment pattern, including ulcerative colitis signaling in the AD–IBD (*p*-value = 1.23 × 10^−7^) [28], mature B-cell differentiation in the AD–RA (*p*-value = 2.31 × 10^−8^) [21] and granulocyte differentiation (*p*-value = 2.23 × 10^−6^) in the AD–vitiligo [29] comparisons. In addition, no skin-related enriched term was observed in the AD–vitiligo comparison, given the relatively distinct pathogenic cutaneous mechanisms in both diseases [16]. Similarly to the gene-level analyses, we observed large overlaps across all traits including 57 terms mapped across at least two comparisons (AD–IBD: 23.1%, AD–RA: 48.2%, AD–vitiligo: 10.6%), while 38 terms were shared across all 3 comorbidities, involving T cell and leukocyte differentiation, immune system development, regulation of hemopoiesis and cell activation (Table S3).

Tissue enrichment analysis supported the notion of widespread patterns of inflammation across bivariate analyses. More specifically, the top prioritized tissue across all analyses was whole blood, while similar estimates were derived for spleen, lung and Epstein–Barr virus–transformed lymphocytes (Figure S5).

### 2.4. Multi-Trait Colocalization Analysis

Multi-trait colocalization analysis using HyPrColoc identified 22 pleiotropic loci between AD and at least one comorbidity at a posterior probability (PP) ≥ 0.6 (Table 2). In 4 of these loci, we prioritized a single causal variant for the colocalization explaining ≥ 80% of the colocalization signal, including the rs72837826 explaining 90.39% of the colocalization between all traits, the rs212389 colocalized between AD and RA (PP.SNP = 0.806), the rs175714 explaining 81.9% of the colocalization signal between AD and RA and the rs12324931 explaining 100% of the PP association between AD and IBD. Moreover, 4 loci were colocalized with all traits under study (Table 2). A complete list of all colocalization results is presented in Table S4. We next calculated the 95% credible set of SNPs in every colocalized locus and uploaded them to the VEP web platform to predict their functional consequences on gene transcripts, protein sequences, and regulatory regions (Table S5). The 177 set of SNPs was mapped in 47 unique genes across all colocalized loci, prioritizing genes associated with all traits, including *PTPN22* (shared among 2 bivariate comparisons in the MAGMA analysis (Figure 2b)), *BCL2L11*, the long non-coding RNA *LINC00824,* and the established pleiotropic *SH2B3*-*ATXN2* locus (Table 2).

**Figure 3:**
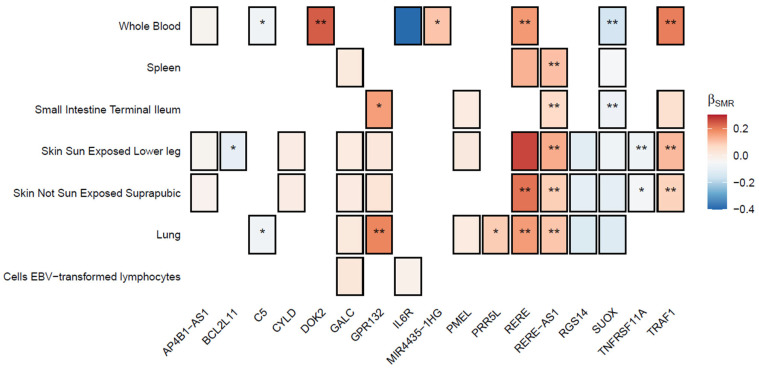
Summary-based mendelian randomization estimates from VEP-prioritized genes (Table S5) in AD-related tissues [7,30]. White tiles indicate the absence of gene cis-eQTL associations in the tissue. Tiles with a single asterisk (“*”) denote nominally significant gene-trait associations (*p*-value ≤ 0.05) with *p*-value_HEIDI_ ≥ 0.05, while two asterisks (“**”) denote significant gene-trait associations (*p*-value ≤ 0.001) with *p*-value_HEIDI_ ≥ 0.05. A complete list of all results is presented in Table S6.

Finally, we examined the causal association of genetically regulated gene expression of all prioritized, protein-coding genes in AD risk using summary-based Mendelian Randomization (SMR) (Figure 3). In total, 50 gene-trait association tests were performed, resulting in a *p*-value threshold of 0.001 after Bonferroni correction. The antisense transcript of *RERE*, *RERE-AS1*, was identified in 5/7 tissues under study, reporting consistent associations with increased AD risk, with the largest effect observed in sun-exposed skin (β, 95% CI: 0.134, 0.06–0.208, *p*-value = 3.5 × 10^−4^). Positive associations were also observed for *DOK2* in whole blood (β, 95% CI: 0.235, 0.102–0.368, *p*-value = 5.1 × 10^−4^), *GPR132* in lung (β, 95% CI: 0.188, 0.009–0.286, *p*-value = 1.5 × 10^−4^) and *RERE*, alongside *TRAF1* in skin and whole blood tissues (Figure 3). Contrastingly, genetically predicted overexpression of *SUOX* in whole blood (β, 95% CI: −0.158, −0.219–−0.096), *p*-value = 5 × 10^−7^) and small intestine (β, 95% CI: −0.085, −0.12–−0.05, *p*-value = 1.33 × 10^−6^) as well as *TNFRSF11A* in sun-exposed skin (β, 95% CI: −0.078, −0.123–−0.034, *p*-value = 5.6 × 10^−4^) reported protective effects in AD risk (Table S6).

### 2.5. Bidirectional Causal Relationships

**Figure 4:**
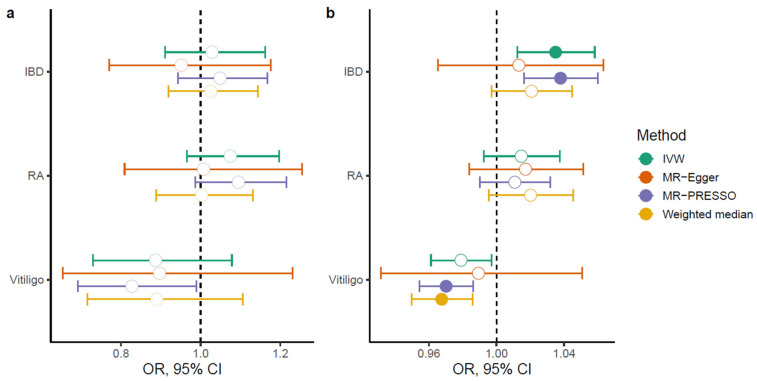
Mendelian randomization estimates when (**a**) atopic dermatitis was set as exposure, and (**b**) atopic dermatitis was set as outcome. Filled nodes refer to significant results (*p*-value ≤ 0.016). Abbreviations: IBD, inflammatory bowel disease; RA, rheumatoid arthritis; IVW, inverse-variance weighted; OR, odds ratio; CI, confidence intervals.

Detailed statistics for all instrumental variables (IVs) used in the bidirectional Mendelian Randomization (MR) analyses are presented in Table S7. Genetic liability to AD reported null results for IBD (odds ratio (OR), 95% confidence intervals (CI): 1.028, 0.911–1.161), RA (OR, 95% CI: 1.07, 0.964–1.197) and vitiligo (OR, 95% CI: 0.886, 0.729–1.078) under the inverse-variance weighted (IVW) model (Figure 4a), showing evidence for significant heterogeneity (Table S8) and non-significant estimates for horizontal pleiotropy through the MR-Egger intercept (*p*-value_IBD_ = 0.38, *p*-value_RA_ = 0.508, *p*-value_vitiligo_ = 0.931; Table S9). MR pleiotropy residual sum and outlier (MR-PRESSO) test identified 5 outliers for IBD, 1 for RA and 2 outliers for vitiligo (Table S10), whereas exclusion resulted in a nominally significant association for vitiligo (OR, 95% CI: 0.827, 0.691–0.989), *p*-value = 0.038; Figure 4a).

In contrast, using AD as the outcome resulted in the identification of causal relationships for IBD and vitiligo, albeit with increased heterogeneity (Table S8). Genetic liability to IBD was associated with increased risk for AD using IVW (OR, 95% CI: 1.03, 1.01–1.05, *p*-value = 0.0023), with similar estimates in sensitivity analyses (Figure 4b) and no evidence for horizontal pleiotropy (*p*-value_IBD_ = 0.595, Table S9). Exclusion of 5 outliers with MR-PRESSO (Table S10) did not significantly alter the results (Figure 4). Despite the genetic liability to vitiligo not being associated with AD risk through IVW, weighted median (OR, 95% CI: 0.967, 0.949–0.985, *p*-value = 0.0005) and MR-PRESSO analyses excluding 2 outliers (OR_,_ 95% CI: 0.97, 0.954–0.986, *p*-value = 0.0003) showed significant protective associations with AD. Genetic liability to RA was not associated with AD risk under any analysis (Figure 4b).

## 3. Discussion

Despite distinct pathogenic frameworks of autoimmune diseases and AD, accumulating epidemiological evidence suggests overlapping genetic architectures and pleiotropic effects. To elucidate the heritable basis of the observed multimorbidity, we scrutinized genome-wide data to examine shared genetic risk between AD and three autoimmune diseases, namely IBD, RA, and vitiligo. The diversity of the selected disorders enabled us to probe whether shared genetic risk with AD reflects broad immune dysregulation or is confined to specific autoimmune subtypes. We used complementary approaches, spanning from global genetic correlations and bidirectional MR to colocalization analyses to disentangle shared genetic liability from disease-specific effects. Compared to previous cross-trait meta-analyses in allergic diseases [9] and autoimmune comorbidities [17], we focused on statistical pleiotropy through pleiotropic analysis under composite null hypothesis (PLACO) to determine whether the same SNPs may contribute to multimorbidity. Our findings reveal that a significant proportion of this pleiotropic heritability is mapped on inflammatory pathways, providing a mechanistic rationale for overlapping therapeutic strategies between AD and autoimmune diseases.

The directionality of these relationships was investigated using bidirectional MR. We found no evidence for a causal effect of genetic liability to AD on any of the autoimmune diseases examined (Figure 4), suggesting that the observed comorbidity is unlikely to reflect AD as an upstream initiator of systemic immune activation. Contrastingly, genetic liability to IBD was positively associated with AD risk (Figure 4b), corroborating prior observational links for increased prevalence of AD in patients suffering from IBD [31,32]. We failed to replicate the results of Meisinger and Freuer reporting significant associations in the inverse direction [33]; nevertheless, we leveraged a larger number of independent IVs from more recent, larger GWAS studies in European individuals. Interestingly, genetic liability to vitiligo was associated with a significant protective effect on AD risk via MR-PRESSO (Figure 4b) after the exclusion of 2 outliers, opposing epidemiological data reporting increased prevalence of AD in individuals suffering from vitiligo [16]. We speculate that the discrepancy between our MR findings and epidemiological data reflects differences in the immune context. Vitiligo is characterized by a T helper (T_H_)1/T_H_17–dominated inflammatory milieu [34], whereas AD exerts a T_H_2-skewed inflammatory response in pediatric and acute disease stages [7]. Since T_H_1 and T_H_2 display antagonistic immune responses, genetic liability toward a T_H_1-dominant profile observed in vitiligo may reduce susceptibility to a T_H_2-mediated AD. Indeed, more than 80% of AD cases in the discovery GWAS [7] employed in our study were of pediatric or adolescent onset, reflecting the acute, T_H_2-driven phase [34], thus explaining the protective effect of vitiligo in AD risk (Figure 4b). Contrastingly, observational studies reporting positive comorbidity [10,16] largely capture later AD stages or shared environmental triggers not accounted for in our genetic analyses. Nevertheless, the MR estimates were of modest size (Figure 4), implying low statistical power to detect such associations.

Although global genetic correlation estimates between AD and 2/3 autoimmune diseases were non-significant, the genome-wide pleiotropy scan revealed 113 shared independent, non-overlapping risk loci across all pairwise comparisons (Figure 2b). Given the predominant enrichment of all GWS pleiotropic variants in intronic regions (Figure 2a), we speculated that gene-level analyses using a ±0 kb window could inform us on some aspects of the shared genetic liability. Indeed, we identified 13 genes consistently implicated in all pairwise comparisons, holding a well-established role in immune dysregulation (Figure 2b). For instance, the OXL40 ligand encoded by the *TNFSF4* gene and the decoy receptor 3 (DcR3) encoded by *TNFRSF6B* modulate T cell differentiation through interaction with antigen-presenting cells, where *TNFSF4* signaling contributes to skin barrier impairment and intense pruritus stimulating effector T cell proliferation [35], while *TNFRSF6B* induces a skewed T helper (T_H_)2 polarization of dendritic cells [36]. Notably, *TNFRSF6B* and *CLEC16A*, a less characterized gene in AD pathogenesis, have shown consistent associations with the disease across European and Asian ancestries, enhancing their critical role as immunomodulatory agents in the cutaneous disorder [37]. Our genome-wide pleiotropy study further identified a gene cluster on the 4q27 chromosome region, encompassing *KIAA1109*, *IL2*, *IL21*, and *ADAD1* genes, reporting a strong literature support for association with most autoimmune diseases and AD [38,39,40]. To fine-tune these associations, we performed multi-trait colocalization followed by integration of GWAS data with *cis*-expression quantitative trait loci (eQTLs) in AD-relevant tissues [30] using SMR (Figure 3). We observed significant associations for *DOK2*, *GPR132*, *RERE* and its antisense transcript *RERE-AS1*, *SUOX*, *TNFRSF11A*, and *TRAF1* in all prioritized tissues. Some of these genes have been previously associated with AD, with the exemplars of *SUOX*, a central regulator in inflamed skin tissues [41], *GPR132*, a transmembrane receptor with suggestive evidence for association with IBD, and *IL6R*, an established risk gene for AD [42]. However, our SMR analysis was based on bulk *cis*-eQTL gene expression, hindering regulatory effects that are highly dynamic across disease stages, severity, and immune cell subsets in AD, similarly to all complex traits. In particular, the transition from the T_H_2-skewed acute phase toward a chronic, T_H_1/T_H_17-dominant profile of AD may reflect shifts in cellular context that also track with disease severity and the multimorbidity onset. Capturing such context-dependent regulation is essential for understanding how pleiotropic genes operate, since their effects are likely to be temporally restricted and cell-type specific. Future work can address these aspects through single-cell techniques, enabling the identification of cell-specific *cis*-eQTL effects, and longitudinal datasets capturing stage-specific immune changes to resolve context-dependent effects and refine the mechanistic interpretation of shared loci. In addition, the overlap between MAGMA (Figure 2b) and SMR (Figure 3) prioritized gene lists was limited. This divergence is likely attributed to the fundamental methodological differences of each analysis, where MAGMA identifies gene-level enrichment based on SNP-wide aggregation of association statistics, while our SMR analysis hypothesized that pleiotropic variants exert their role through *cis*-regulated transcript abundance. While each method captures different dimensions of gene functions, both approaches converged on disrupted immunomodulatory mechanisms as exemplified through gene-set analyses (Table S3) and tissue enrichment results (Figure S5), suggesting different pleiotropic mechanisms at the gene level connecting to common pathways as discussed elsewhere [43].

There are some constraints to our study. GWAS summary statistics originated from individuals of European ancestry (Table 1), hence restricting the generalizability of our findings to other ancestries. The limited generalizability of our findings in non-Europeans is further suggested by the ancestry-specific mechanisms of AD, exerting a T_H_1-skewed inflammation in Asians [30]. Similarly, our analysis did not account for rare variants and SNPs mapped in the MHC, overlooking potential associations, especially regarding AD, where rare protein-coding variants explain a significant proportion of the observed heritability [44]. Our analyses were also restricted to identifying pleiotropy through statistical approaches, thus incapable of distinguishing between true pleiotropy and biological pleiotropy [45]. Fourth, although autoimmune traits were selected based on strong epidemiological links and immunopathological relevance to AD [10], our approach inherently excludes other conditions that may share genetic architecture with AD. To mitigate this, we deliberately chose diseases spanning mucosal, systemic, and organ-specific autoimmunity to maximize biological diversity and pleiotropic discovery potential. Additionally, the multi-layered evidence provided through statistical pleiotropy, multi-trait colocalization, and functional investigation can be applied to any pairwise comparison to future studies of multimorbidity. Lastly, our reliance on available *cis*-eQTL expression data in AD-relevant tissues obscures putative novel pleiotropic associations of highlighted genes (Table S5) in different, non-AD-related tissues; expanding future analyses to single-cell and disease-contextual regulatory data will be critical for resolving cellular and systemic specificity of shared genetic signals.

## 4. Methods

### 4.1. Data Sources

We gathered GWAS summary statistics from publicly available sources including the GWAS Catalog [23]. To maximize statistical power, we prioritized studies with the largest number of cases and controls in European participants. Details for every GWAS summary statistic used in the current study are provided in Table 1. All data received approval from the appropriate ethics committees, with written and informed consent from every participant. Collected data occurred over the course of November 2024, and the analysis started in November 2024. *Cis*-eQTL summary data were downloaded from the genotype-tissue expression (GTEx) v8 dataset of the GTEx Portal [46]. All analyses and reported results were aligned to the human genome assembly GRCh37/hg19.

### 4.2. Genetic Correlation

To compute the global genetic correlation estimates between AD and autoimmune diseases, we used linkage disequilibrium score regression (LDSC) [47]. Variants were restricted to the well-imputed HapMap3 SNPs, and linkage disequilibrium (LD) scores were precomputed from the European ancestry samples *(n* = 503) from the 1000 Genomes Project, excluding low-frequency variants with minor allele frequency (MAF) ≤ 1%. A Bonferroni-corrected *p*-value threshold of 0.016 (0.05/3) was set as the significance threshold.

### 4.3. Pleiotropy Analysis

To identify potential pleiotropic associations at the SNP level, we performed PLACO [48]. In particular, PLACO decomposes the null hypothesis into three distinct sub-null hypotheses, where the variant under study may be associated with at most one trait, while the alternative hypothesis refers to the association of the SNP with both traits. All GWAS summary statistics were aligned to the same reference allele with the AD GWAS [7] to ensure robust associations. Variants were filtered for (i) common SNPs (MAF ≥ 1%), (ii) mapped outside the major histocompatibility complex (MHC) (chr6:25 × 10^6^–35 × 10^6^; GRCh37) to avoid complex LD patterns, and (iii) biallelic, unique variants. Variants with extremely large effect sizes (Z-score^2^ ≥ 80) were excluded from the analysis following the recommendation of the PLACO developers [48], since large effect SNPs are often statistical outliers disproportionately influencing regression estimates and leading to spurious results [47]. A genome-wide significance threshold of 5 × 10^−8^ was adopted to identify pleiotropic SNPs.

### 4.4. Functional Annotation

Summary statistics were then imported to FUMA web platform to define candidate pleiotropic loci [49]. The European reference panel of the 1000 Genome Project, Phase 3, was used to compute LD scores. A two-step LD-based clumping process was executed using PLINK v1.9 to define genomic regions. In the first step, an r^2^ ≥ 0.6 threshold is applied to define genomic risk loci, clumping independent significant SNPs. Next, a stricter r^2^ ≥ 0.1 threshold is employed to declare lead SNPs. Functional annotation of genome-wide significant (GWS) variants was performed via ANNOVAR (v2016Dec05) [50] to categorize based on their location in accordance with genes and their coding function. Positional mapping is executed based on the genomic position of the SNPs or on their consequences on gene functions. Statistical significance was set to a 0.0015 threshold after applying Bonferroni correction (0.05/(11 functional categories × 3 bivariate comparisons)).

Gene-level, gene-set, and tissue enrichment analyses were executed using MAGMA v1.10 software to determine pleiotropic genes and biological pathways, a multiple regression-based framework that evaluates joint SNP effects on gene-level associations [51]. SNP to gene mapping was conducted using predefined gene boundaries from the Ensembl v110 platform using a symmetric ±0 kb window, and gene-wide *p*-values were derived by aggregating SNP-level statistics accounting for LD structure. Competitive gene-set analysis was conducted with established pathways from the molecular signatures database (MSigDB) v2023.1 human collections. Tissue-specific expression analysis was conducted using GTEx v8 data to identify tissues enriched for pleiotropic genetic signals. Results from MAGMA were corrected for multiple comparisons using the Bonferroni method in a pairwise manner.

### 4.5. Multi-Trait Colocalization Analysis

Multi-trait colocalization analysis was performed using HyPrColoc with the default parameters, a Bayesian clustering algorithm that enables fast and accurate estimations of colocalization probabilities in a genomic region across a large number of binary and continuous traits [52]. Colocalization analyses were performed at all non-overlapping pleiotropic loci, defined as a ±250 kb around the lead variant of each pairwise comparison. A PP threshold of 0.6 was adopted to declare strong evidence for colocalization. For each significant colocalization, we gathered the 95% credible set of SNPs, consequently annotating their functional role and gene mapping through the Ensembl variant effect predictor (VEP) web platform [53]. The summary-based Mendelian randomization (SMR) was finally employed to dissect the causal effects of pleiotropic genes identified through VEP in AD risk, using the top *cis*-eQTL to evaluate the association of each probe and AD [54]. The heterogeneity in dependent instruments (HEIDI) test was employed to distinguish pleiotropy from linkage, using SNPs in a *cis*-eQTL region. We used 7 previously identified AD-relevant tissues, including spleen, lung, whole blood, small intestine terminal ileum, sun-exposed and not sun-exposed skin, and Epstein–Barr virus–transformed lymphocytes [7,30]. Significance threshold was set at a Bonferroni-corrected threshold of *p*-value ≤ 0.001, while the *p*-value threshold regarding the HEIDI test was set to *p*-value_HEIDI_ ≥ 0.05.

### 4.6. Bidirectional Causal Relationships

Finally, we executed two sample bidirectional MR analyses to investigate causality between genetic liability to AD and to autoimmune diseases under study. IVs were selected based on the 3 MR hypotheses, including (i) robust association with the exposure, (ii) independence with any confounders between the exposure and the outcome, and (iii) lack of horizontal pleiotropy. Independent IVs were selected based on a *p*-value threshold of ≤5 × 10^−8^ and clumped at an r^2^ = 0.001 threshold using the European 1000 Genomes Project Phase 3 reference data. Variants were harmonized between the exposure and the outcome to ensure that the IV calculations referred to the same allele. For each IV, we calculated the F-statistic as the squared beta divided by the squared standard error (β^2^/se(β)^2^); a threshold of 10 was used to declare strong IVs and reduce any risk of weak instrument bias [55].

The IVW method was used as the primary model, combining Wald ratio estimates of each IV to perform an inverse-variance weighted analysis [56]. The level of heterogeneity of the IVW estimates was quantified using Cochran’s Q statistic [57]. We next performed sensitivity analyses, including the MR-Egger, an extension to the IVW approach assuming a linear relationship between the exposure and the outcome of every test, accounting for horizontal pleiotropy [58]. The intercept of the MR-Egger test was used to detect horizontal pleiotropy. In addition, the weighted median estimator is a robust method that provides a consistent estimate of the causal effect if at least 50% of the weight comes from valid instrumental variables [59]. Finally, to account for non-detectable violations of the MR assumptions and identify outliers, we performed the MR-PRESSO analysis, recalculating IVW estimates after excluding potential outliers [60]. A Bonferroni-corrected significance threshold of 0.016 was set to declare significant results accounting for multiple comparisons.

## 5. Conclusions

In conclusion, we identified 113 non-overlapping pleiotropic loci jointly associated with AD and autoimmune diseases with discrete immunopathological mechanisms. Integration of multiple gene prioritization algorithms pinpointed to discrete genes, nevertheless converging on shared immune-related pathways that could serve as novel therapeutic targets. Our findings stress the role of inflammation as a unifying axis linking epithelial and autoimmune disorders, providing evidence for differential pleiotropic mechanisms across disease domains. This pleiotropy-informed framework lays the foundation for identifying shared mechanisms at the gene and pathway level, with potential implications for drug repositioning in AD and immune-mediated comorbidities.

## Figures and Tables

**Table 1:** Summary of genome-wide association studies used in the study.

Disease	Sample Size	Cases	Controls	Ancestry	Authors
AD [7]	864,982	60,653	804,329	European	Budu Aggrey et al., 2023
IBD [24]	59,957	25,042	34,915	European	de Lange et al., 2017
RA [25]	97,173	22,350	74,823	European	Ishigaki et al., 2022
Vitiligo [26]	44,266	4680	39,586	European	Jin et al., 2016

Abbreviations: AD, atopic dermatitis; IBD, inflammatory bowel disease; RA, rheumatoid arthritis.

**Table 2:** Multi-trait colocalization results using HyPrColoc.

Genomic Coordinates	Colocalized Traits	PP	SNP	SNP.PP	Mapped Genes	AD GWAS *p*-Value
chr1:7900638-8739302	RA, vitiligo, AD	0.7127	rs301799	0.3816	*RERE*, *RERE-AS1*	1.43 × 10^−7^
chr1:114053808-114573318	IBD, RA, vitiligo, AD	0.8452	rs6679677	0.7535	*PTPN22*, *AP4B1-AS1*, *RSBN1*, *PHTF1*	7.18 × 10^−6^
chr1:154170087-154675456	RA, AD	0.8875	rs12133641	0.5913	*IL6R*	1.72 × 10^−21^
chr2:111658567-112183001	IBD, RA, vitiligo, AD	0.9422	rs72837826	0.9039	*BCL2L11*, *MIR4435-2HG*, *ENSG00000295185*	2.82 × 10^−6^
chr5:176544191-177044191	IBD, AD	0.7366	rs12654812	0.6639	*RGS14*	7.66 × 10^−8^
chr6:90680513-91226768	IBD, vitiligo, AD	0.7597	rs17513531	0.1207	*BACH2*	3.85 × 10^−10^
chr6:106417535-106917535	RA, AD	0.8917	rs9372120	0.34	*ATG5*, *U4*, *ENSG00000303838*, *RN7SL47P*	1.08 × 10^−7^
chr6:159222295-159739791	RA, AD	0.8782	rs212389	0.8069	*ENSG00000285492*, *TAGAP-AS1*, *TAGAP*	1.82 × 10^−11^
chr8:21519432-22019432	IBD, AD	0.9431	rs56094005	0.5426	*DOK2*	9.35 × 10^−9^
chr8:126341392-126858642	RA, AD	0.7486	rs28550378	0.3259	*ENSG00000302425*	3.91 × 10^−15^
chr8:129302491-129802540	IBD, RA, vitiligo, AD	0.7118	rs938650	0.5337	*LINC00824*, *ENSG00000298619*, *ENSG00000298640*	3.75 × 10^−5^
chr9:123441237-123941237	RA, AD	0.6172	rs1930785	0.097	*TRAF1*, *C5*, *PHF19*, *ENSG00000306516*	3.41 × 10^−5^
chr11:36187868-36735919	IBD, AD	0.6701	rs28520436	0.316	*PRR5L*	1.22 × 10^−24^
chr11:118492800-118992800	RA, AD	0.9434	rs74541740	0.1599	*ENSG00000306274*, *ENSG00000306318*	2.19 × 10^−10^
chr12:56134804-56694632	RA, AD	0.984	rs705699	0.6027	*CDK2*, *RAB5B*, *PMEL*, *SUOX*	3.31 × 10^−9^
chr12:111634608-112257756	IBD, RA, vitiligo, AD	0.9443	rs3184504	0.7639	*SH2B3*, *ATXN2*	1.56 × 10^−8^
chr14:75731856-76231856	RA, AD	0.9868	rs175714	0.8197	*ENSG00000297883*	1.44 × 10^−7^
chr14:88176297-88676297	IBD, AD	0.9252	rs4462528	0.4761	*GALC*, *SHLD2P2*	5.99 × 10^−6^
chr14:105273663-105773663	IBD, AD	0.7636	rs7147439	0.5047	*LINC02298*, *GPR132*, *ENSG00000307140*	4.71 × 10^−8^
chr16:50763434-51263434	IBD, AD	0.6399	rs12324931	1	*CYLD*	0.000261
chr17:38507789-39007789	IBD, AD	0.8969	rs1358175	0.6417	*ENSG00000279775*	1.99 × 10^−11^
chr18:59759814-60272811	RA, AD	0.9242	rs4574025	0.5308	*TNFRSF11A*	1.48 × 10^−6^

Abbreviations: PP, posterior probability; SNP, single-nucleotide polymorphism; AD, atopic dermatitis; GWAS, genome-wide association study; IBD, inflammatory bowel disease; RA, rheumatoid arthritis.

## Data Availability

The data presented in the study are openly available in the GWAS catalog (https://www.ebi.ac.uk/gwas/).

## Supplementary Materials

The supporting information can be downloaded at: https://www.mdpi.com/article/10.3390/ijms26189124/s1.
