# Supplementary material for: Shared Genetic Architecture Between Atopic Dermatitis and Autoimmune Diseases
Source: Int J Mol Sci. 2025 Sep 18;26(18):9124. doi: 10.3390/ijms26189124 (PMC12470386; doi:10.3390/ijms26189124)

Figure S1. Comparison of Z scores for pleiotropic variants between atopic dermatitis (x axis) and inflammatory bowel disease (y axis). Each node corresponds to a pleiotropic lead variant. Nodes colored in red represent concordant sign, while nodes colored in blue represent discordant sign.

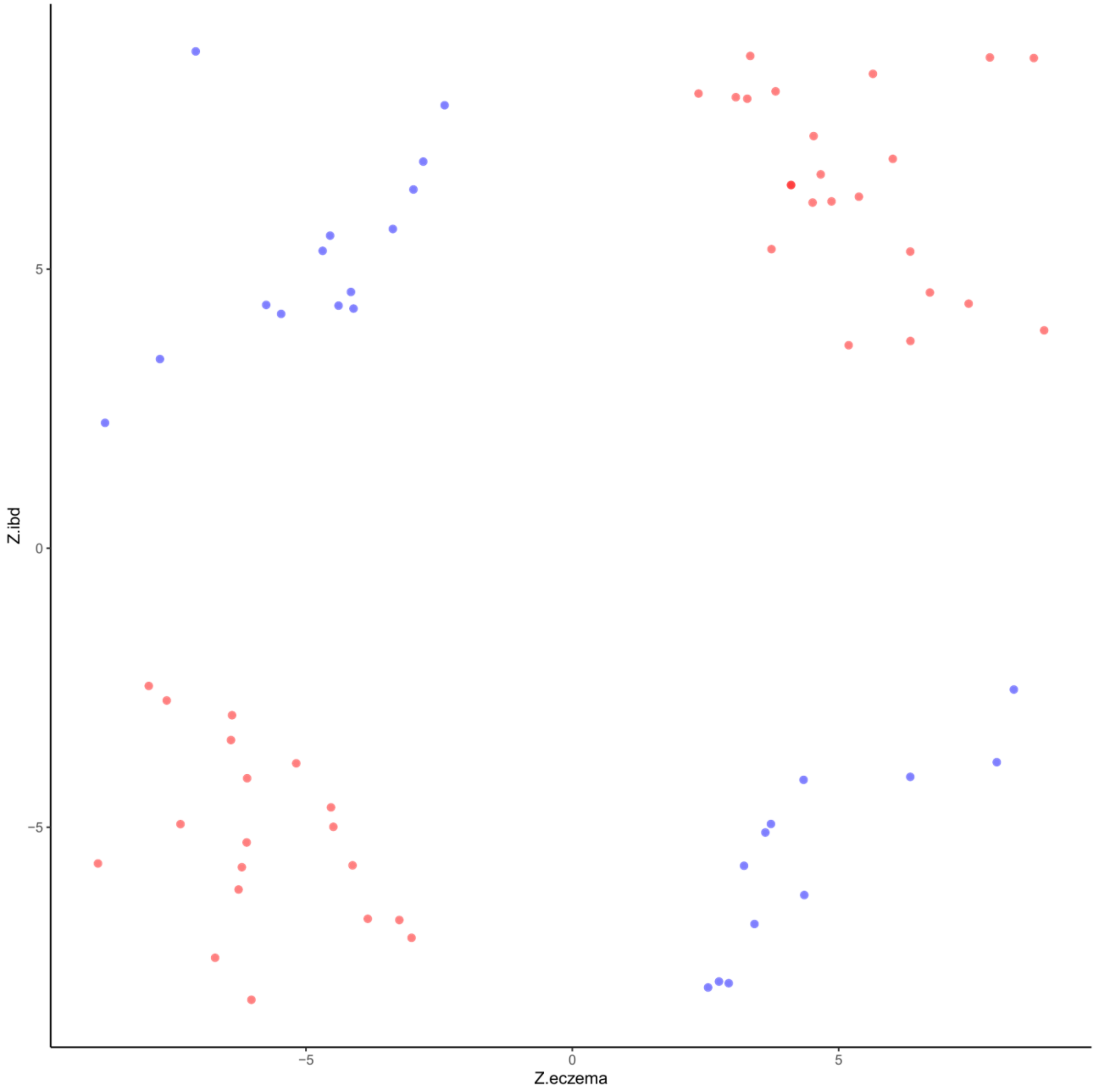

Figure S2. Comparison of Z scores for pleiotropic variants between atopic dermatitis (x axis) and rheumatoid arthritis (y axis). Each node corresponds to a pleiotropic lead variant. Nodes colored in red represent concordant sign, while nodes colored in blue represent discordant sign.

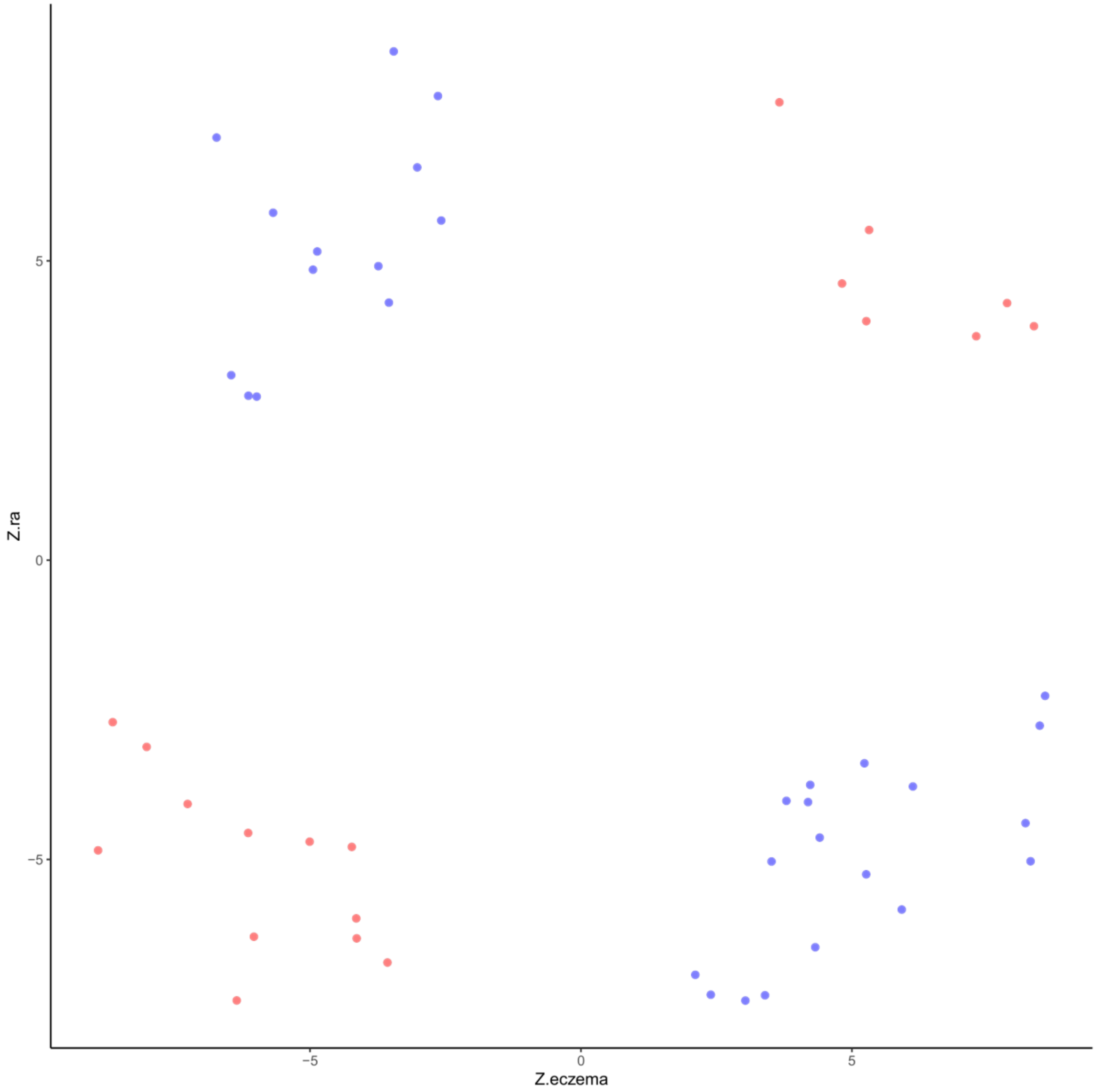

Figure S3. Comparison of Z scores for pleiotropic variants between atopic dermatitis (x axis) and vitiligo (y axis). Each node corresponds to a pleiotropic lead variant. Nodes colored in red represent concordant sign, while nodes colored in blue represent discordant sign.

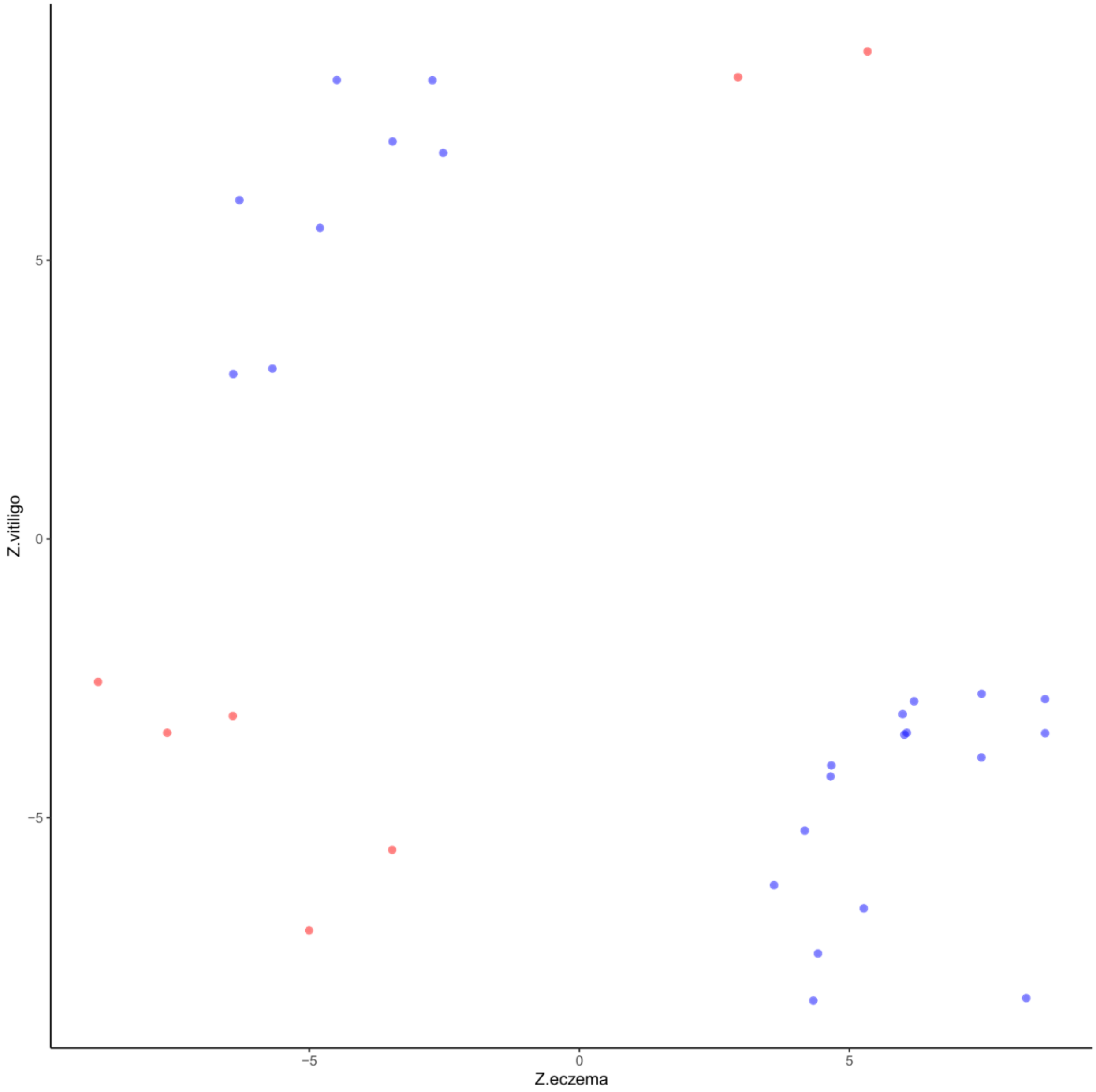

Figure S4. Network plot of shared genes prioritized through MAGMA between each pleiotropic comparison.

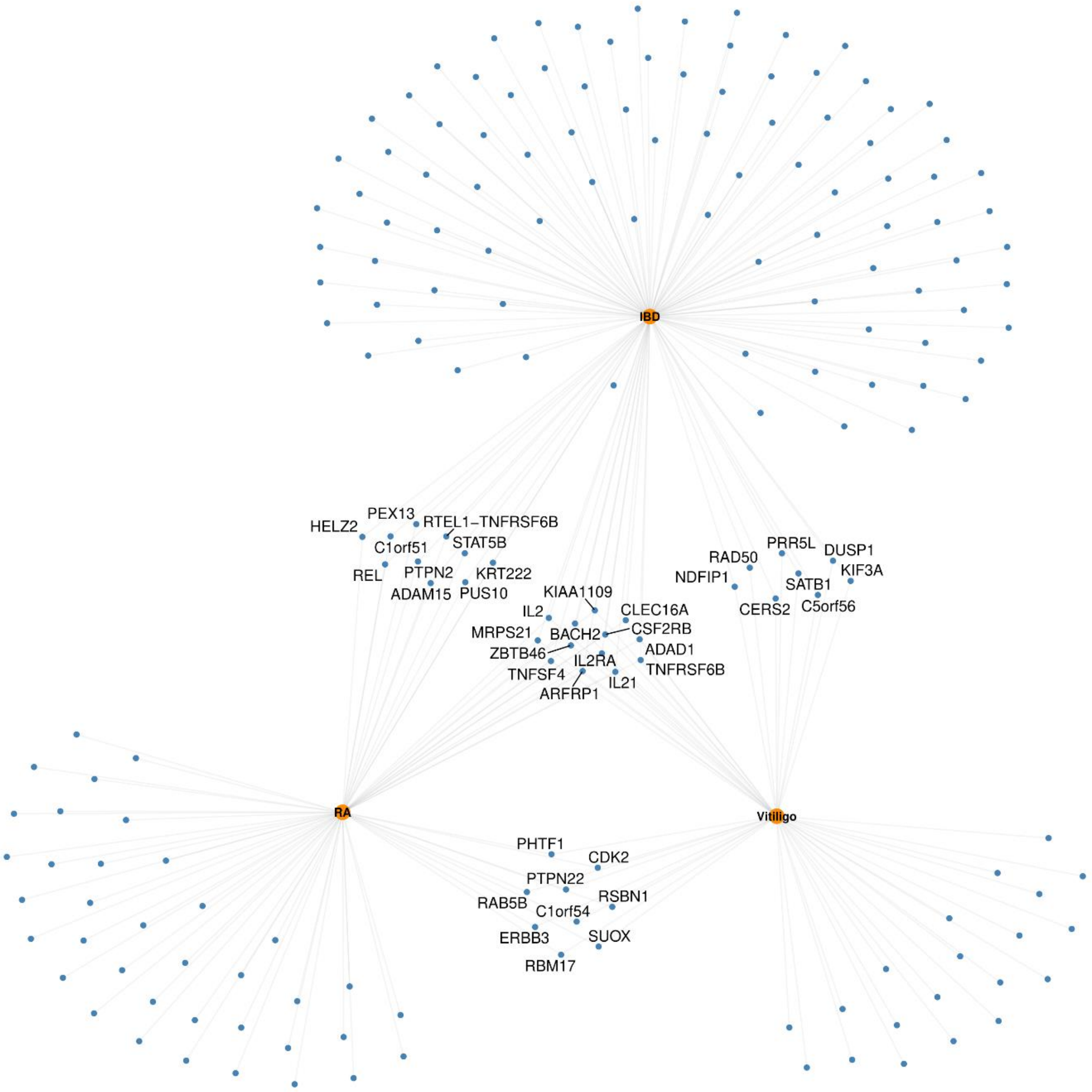

Figure S5. Tissue enrichment analysis for pleiotropic associations between atopic dermatitis and (a) inflammatory bowel disease, (b) rheumatoid arthritis and (c) vitiligo. The black line denotes tissue-specific statistical significance (P-value=0.05/54).

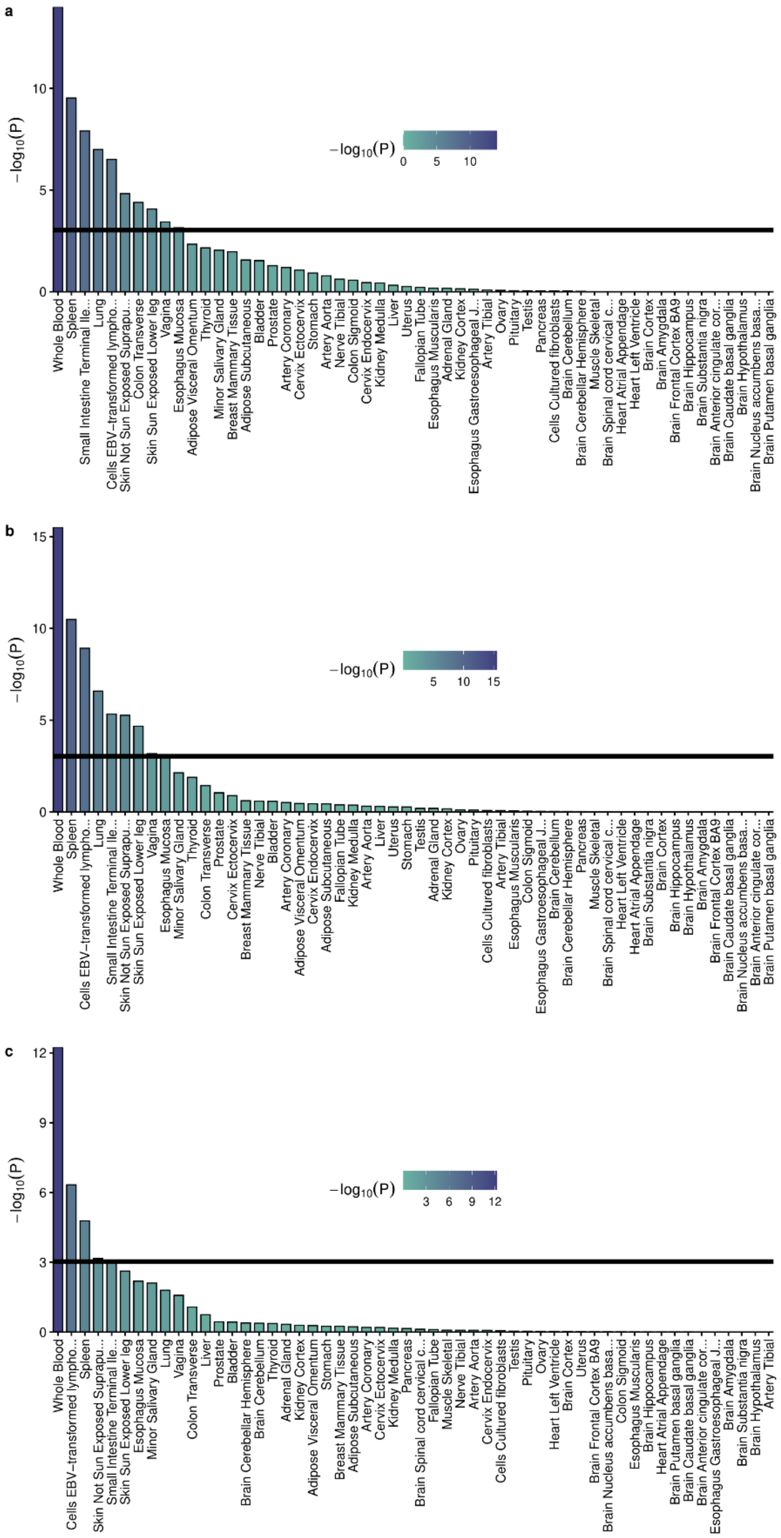

Supplement: Supplementary file 1 [file ijms-26-09124-s001.zip › Supplementary_Figures.pdf]
